# Challenges, knowledge, and skills required for family caregivers of older adults with dementia: a qualitative study in Vietnam

**DOI:** 10.1080/16549716.2025.2526929

**Published:** 2025-07-08

**Authors:** Tran To Tran Nguyen, The Ha Ngoc Than, Tuan Chau Nguyen, Lan Tuyet Duong Vu, Khoa Tri Truong, Truc Thanh Thai, Penelope Schofield, Tuan Anh Nguyen

**Affiliations:** a Department of Geriatrics, University of Medicine and Pharmacy, Ho Chi Minh City, Vietnam; b Department of Geriatrics, Nhan dan Gia Dinh Hospital, Ho Chi Minh City, Vietnam; c Department of Geriatrics and Palliative Care, University of Medicine and Pharmacy at Ho Chi Minh City, Ho Chi Minh City, Vietnam; d Department of Rheumatology, Ho Chi Minh City University Medical Center, Ho Chi Minh City, Vietnam; e Department of Medical Statistics and Informatics, University of Medicine and Pharmacy at Ho Chi Minh City, Ho Chi Minh City, Vietnam; f School of Computing, Engineering and Mathematical Sciences, La Trobe University, Melbourne, Australia; g Department of Psychological Sciences, and Iverson Health Innovation Research Institute Swinburne University, Melbourne, Victoria, Australia; h Sir Peter MacCallum Department of Oncology, The University of Melbourne, Parkville, Victoria, Australia; i Social Gerontology Division, National Ageing Research Institute, Melbourne, Australia; j UniSA Clinical and Health Sciences, University of South Australia, Adelaide, Australia; k Health Strategy and Policy Institute, Ministry of Health, Hanoi, Vietnam

**Keywords:** Anxiety, care burden, gaps, stress, support services

## Abstract

**Introduction:**

Most people with dementia (PwD) receive home care from their family members. Caring for PwD can be challenging; however, many family caregivers do not receive adequate training. The lack of support, knowledge, and skills in dementia care places significant physical and psychological stress on family caregivers.

**Objectives:**

This qualitative study explored the challenges faced by family caregivers in providing care for PwD and identified the knowledge and skills they need for effective caregiving roles.

**Methods:**

In-depth interviews with 20 family caregivers of PwD were conducted face-to-face using a semi-structured questionnaire at the Department of Geriatrics at Nhan dan Gia Dinh Hospital in Ho Chi Minh City. Data analysis was conducted using inductive content analysis. The interview transcripts were coded by two independent researchers and analyzed thematically.

**Results:**

Six key themes emerged: (1) challenges in daily care; (2) strain from behavioral and sleep disruptions; (3) difficulties in seeking help; (4) personal sacrifices of caregivers; (5) emotional burdens associated with caregiving, including comorbidity concerns of PwD; (6) limited dementia knowledge and essential caregiving skills;

**Conclusions:**

The study highlights the need for targeted interventions in dementia care and underscores the necessity of providing educational and support programs for family carers of people with dementia in Vietnam and other low- and middle-income countries. Effective educational programs should cover dementia knowledge, comorbidity management, help-seeking strategies, caregiver self-care, and caregiving skills while also promoting support resources such as respite care and accessible information platforms.

## Paper context

**Main findings:**

Family caregivers of people with dementia in Vietnam face significant challenges in daily care, behavioral management, emotional stress, and insufficient knowledge, exacerbated by cultural and financial barriers to obtaining support.

**Added knowledge:**

This study highlights significant gaps in caregiver education, particularly regarding knowledge of dementia, fall prevention, pressure injury care, and infection management, underscoring the need for targeted training and policy interventions.

**Global health impact on policy and action:**

Strengthening dementia care policies in low- and middle-income countries necessitates integrating caregiver education, establishing support services such as respite care and counseling, and addressing gender-based caregiving disparities to improve the quality of care and the well-being of caregivers.

## Introduction

Globally, dementia is a leading cause of disability among older adults [1] with two-thirds of people with dementia (PwD) residing in low- and middle-income countries (LMICs) [2]. The majority of older PwD receive care at home from family members [3]. While family caregivers play a crucial role in dementia care, they often face significant physical, emotional, social, and financial burdens, and lack the adequate training to provide optimal care [4]. PwD may live four to eight years or longer after diagnosis [5], with caregiving demands progressively increasing as their physical and cognitive health deteriorates. Over time, the need for assistance shifts from instrumental activities of daily living to full personal care, often requiring caregivers to provide up to 10 hours of care per day in the later stages of dementia. This prolonged caregiving can lead to physical exhaustion and increased exposure to behavioral and psychological symptoms, such as aggression, hallucinations, delusions, disinhibitions, and sleep disturbances, all of which contribute to caregiver stress and burnout [6].

Despite these challenges, many caregivers, particularly in Eastern LIMCs and Southeast Asia, have limited access to training, educational resources, and support services [7]. The cumulative burden on family caregivers is substantial, with research indicating that they experience higher levels of stress and depressive symptoms compared to caregivers of individuals with other chronic diseases [8]. The impact of caregiver strain extends beyond personal well-being, potentially affecting the quality of care provided. High levels of stress can result in neglect of the care recipient and, in some cases, verbal or physical mistreatment of individuals with dementia [9].

In Vietnam, a rapidly aging LMIC, is not an exception, with the majority of PwD receiving care at home from their family members [10]. This places a substantial burden on family caregivers, many of whom have limited knowledge about dementia. Previous studies indicate that most caregivers in Vietnam have low dementia literacy [11], with over 90% believing that dementia is a normal part of aging. They also often lack adequate support from the healthcare system and society [12].

Although dementia has been recognized globally as a public health priority since 2017, significant gaps remain in medical and social policies for both Vietnamese PwD and their caregivers [12,13]. Dementia diagnoses are often overlooked in clinical practice, and there are currently limited formal medical policies in place to support PwD or their caregivers. For instance, respite care centers have not been established, and there is a critical shortage of support programs and counseling services for caregivers. These systematic gaps place a considerable strain on caregivers, affecting their well-being and ability to provide quality care [14].

According to the Theory of Caregiver Stress [15] and the Roy Adaptation model [16] contextual stimuli (e.g. behavioral changes in care recipient, family conflicts, and work-related strain) are assessed in relation to the caregivers’ adaptive capacities (e.g.. coping mechanisms and social support). When these stressors exceed the caregiver’s ability to adapt, they can lead to caregivers’ perceived stress and emotional or behavioral responses such as depression and anxiety. A lack of social support, caregiving knowledge, and practical skills diminishes caregivers’ adaptive capacities, thereby contributing to caregivers’ stress and depression [17,18]. Given this, contextual stimuli should be a key focus of interventions aimed at reducing stress and depression of family caregivers of PwD [19].

Understanding the needs of family caregivers of PwD is essential for developing effective interventions and support systems [20]. However, there is a lack of research examining these issues in Vietnam. To address this gap, we conducted a qualitative study to explore caregivers’ challenges and identify the knowledge and skills they require. The findings from this study will inform the development of targeted intervention programs to alleviate caregiver stress, improve their well-being, and enhance the quality of care provided to PwD.

## Methods

### Study design

We conducted a qualitative descriptive study to explore the challenges family caregivers of PwD face and to identify the knowledge and skills they require to provide effective care. Given the explorative nature of this inquiry, a qualitative approach was most appropriate. The study was reported in accordance with the Consolidated Criteria for Reporting Qualitative Research (COREQ) Checklist [21].

### Setting and participants

The study was conducted at the Department of Geriatrics at Nhan dan Gia Dinh Hospital, one of the largest public hospitals in Ho Chi Minh City, Vietnam. Most patients admitted to this hospital were from low to middle-income backgrounds and relied on government health insurance. Patients with dementia in this study were defined as individuals diagnosed with dementia based on DSM-5 receiving care at the Department of Geriatrics. Eligible family caregiver participants included: 1) individuals aged 18 years or older who were capable of independently consenting to participate; 2) primary caregivers of PwD who had been most involved in caring for PwD for at least six months, a sufficient duration for caregivers to experience significant stress and burden [22]. 3) having a score of at least four on the Distress Thermometer (ranging from 0 to 10) in the past week, indicating a high level of stress among family caregivers, as supported in prior research [23,24]; and 4) caregivers who were able to communicate in Vietnamese and willing to participate in the study. The participants were excluded from the study if they met any of the following criteria: 1) the presence of a serious chronic illness, such as metastatic cancer, which could contribute to increased stress and burden [25,26]; 2) vision or hearing impairments, which might hinder the interview process; or 3) cognitive impairment, defined as a Mini-Cog score of less than 4, which could affect the accuracy, depth, and reliability of the data collected [27].

### Procedure and materials

Participants were recruited consecutively. First, a research assistant, who is a general doctor not involved in patient treatment, screened for eligible participants. The assistant then introduced the study to the potential participants and invited them to participate. Face-to-face interviews were conducted at a convenient time for participants. An investigator not involved in treatment at the Department of Geriatrics during the study period undertook the in-depth interviews in a private room at Nhan dan Gia Dinh Hospital. All interviews were audio recorded. Additionally, another member of the research team observed and documented the participants’ postures and emotional expressions during the interviews. A semi-structured questionnaire (Appendix 1) guided the in-depth interview, focusing on the following main topics: 1) the primary challenges of providing care; and 2) the knowledge, skills, and support needed by family caregivers. Real-time participant validation was conducted during the interviews, wherein the interviewer confirmed responses with participants to ensure their perspectives were accurately captured. This immediate clarification helped refine unclear responses, enhancing the accuracy and reliability of the data. After each interview, participants received a gift voucher worth approximately US$10 as compensation for their time. Each interview lasted between 50 and 60 minutes, and each participant was interviewed once. Transcripts were not returned to participants for review feedback.

### Data analysis

The audio-recorded interviews were transcribed verbatim. Data analysis was conducted using inductive content analysis [28,29], which offers a systematic and flexible approach to analyzing textual data, allowing for the identification of patterns, themes, and meanings within the experiences shared by family caregivers of PwD. Codes were generated inductively to represent segments of text, then grouped into categories based on similarities, and finally, related categories were organized into overarching themes. During opening coding, emerging concepts were identified and explored. After each interview, the audio recording was transcribed and analyzed to guide the interview process. Emerging themes and new topics of inquiry were explored in the subsequent interviews. The interview transcripts were then compiled, and relevant text segments were organized by themes. Two independent coders manually coded the transcripts. They then discussed, refined, and resolved differences through consensus to develop major themes and categories. Microsoft Excel was used for data management, coding, and thematic identification.

The Vietnamese research team initially analyzed the data in Vietnamese. Subsequently, two mentors in Australia reviewed the findings to refine higher-order categories and assess data saturation. Finally, the themes and categories related to the information, skills, and support needed by family caregivers of people with dementia were formulated.

## Results

Of the 23 eligible family caregivers invited to participate in the study, three declined due to a preference for not sharing their personal experiences. Consequently, 20 family caregivers completed the in-depth interviews. The majority of our participants were middle-aged and predominantly female (90%). All caregivers lived with the individuals they cared for and devoted a substantial amount of time to caregiving, averaging over nine hours per day. Participant characteristics are summarized in Table 1.

**Table 1.** Characteristics of Vietnamese caregivers of PwD.

Characteristics	N = 20n (%)
Age	55.65 ± 10.14 (range 36–70)
Gender	
Female	18 (90)
Male	2 (10)
Educational level	
Primary school	3 (15)
Secondary school	15 (75)
High school	0
Tertiary school/University/Post-graduate	2 (10)
Marital status	
Widow/Divorce	2 (10)
Having a partner	12 (60)
Single	6 (30)
Relationship with patient	
Spouse	2 (10)
Son/Daughter	14 (70)
Son/Daughter in law	1 (5)
Relatives	3 (15)
Occupation	
Farmer	1 (5)
Worker	4 (20)
Business	6 (30)
Officer	2 (10)
Housewife	5 (25)
Others	2 (10)
Living with family (yes)	20 (100)
Having a chronic disease	9 (45)
Comorbidity/Chronic disease	
Hypertension	2 (10)
Diabetes	2 (10)
Ischemic heart disease	1 (5)
Dyslipidemia	2 (10)
Venous insufficiency	2 (10)
Osteoarthritis	3 (15)
Osteoporosis	2 (10)
Multimorbidity	2 (10)
Duration of care (years)	5.2 ± 4.17 (1–15)
Average care hours per day	
7–9	1 (5)
>9	19 (95)
Average income per month (million VND)	
<5 (<200 USD)	13 (65)
≥5 (≥200 USD)	7 (35)

We identified six major themes from the data. Table 2 presents the coding trees for the challenges in providing care for people with dementia.

**Table 2.** Coding trees for challenges in providing care for people with dementia.

Themes	Categories	Sample of codes
Challenges in daily care	Feeding difficulties	Irregular eating, depending on the mood Forgets how to chew and swallow
	Managing incontinence	Frequent episodes of uncontrolled urination/defecation Rarely calls for assistance
	Challenges of hygiene for bedridden individuals	Sole caregiver responsible for all hygiene tasks Lifting the person during a bath is physically demanding
Behavioral strain	Aggression from PwD	Physical and verbal aggression toward the caregiver Destructive behaviors such as tearing clothes
	Sleep disruptions	Sleep deprivation due to nighttime walking of PwD
Difficulties in seeking help	Lack of family help	Feeling alone with caregiving duties
	Cultural gender roles in caregiving	Male family members are perceived as unsuitable for caregiving
	Hiring external caregivers is not an option	Lack of trust in outsiders to provide adequate care Unable to afford paid caregiving
Sacrifices of caregivers	Health sacrifices	Neglecting personal health because of caregiving duties Physical health declining due to stress
	Loss of personal time and work	All the time and energy dedicated to caregiving
Emotional challenges in caregiving	Frustration and stress	Emotional exhaustion from caregiving with minimal improvement
	Guilt and self-doubt	Self-blame for worsening condition of PwD
	Fear and anxiety about the comorbidity of PwD	Fear of falls and medical complications Frequent hospitalizations of PwD
Limited dementia knowledge and essential caring skills	Lack of awareness about dementia	Misconceptions equating dementia with mental illness
	Relying on personal experiences or the internet	Providing care based on personal judgment or informal sources
	Essential caring skills for PwD	Need for practical strategies to manage behavioral symptoms

### Theme 1: family caregivers faced challenges in assisting PwD with activities of daily living

#### Feeding PwD was difficult

Participants expressed frustration over the challenges of feeding their loved ones. Some PwD were uncooperative during meals: ‘*When I fed him, he would not open his mouth*’ (Caregiver [C] 3, age 61, daughter). Others exhibited difficulties such as sucking on the food instead of chewing or spitting it out. Some caregivers also struggled with their loved ones having lost their teeth and being unable to get dentures. To address these challenges, caregivers adopted various strategies. Some prepared softer foods: ‘*When he refused to eat, I made soup or soft potatoes for him*’ (C1, age 70 yrs, wife). Others divided meals into smaller portions, frequently changed the menu, or prepared care recipient’s favorite dishes. In one case, a care recipient required tube feeding: ‘*The doctor said my mom developed pneumonia due to food aspiration. From that point on, the doctor recommended tube feeding. She has now been on on feeding tube for three months*.’ (C20, age 66, daughter).

#### Managing incontinence was a physically demanding and emotionally taxing task for caregivers

Many reported that managing incontinence in PwD significantly increased their caregiving burden. Care recipients often soiled their clothing or bedding, making hygienic maintenance particularly challenging: ‘*My mom urinates at any time. Changing her clothes and cleaning the bed is very tough*.’ (C2, age 65, daughter). Some PwD resisted wearing diapers, further complicating care. In response, some caregivers resorted to using restraints to manage incontinence-related behaviors: ‘*Sometimes, I have to tie her hand to the bed to prevent her from tossing the diapers*.’ (C11, age 41, relative). The emotional toll of incontinence care extended beyond primary caregivers, with some noting that other family members distanced themselves from their loved ones due to the associated challenges. Additionally, constipation was another common issue among PwD, further exacerbating caregivers’ challenges.

#### Maintaining hygiene for bedridden PwD posed significant challenges for family caregivers

Many care recipients with advanced dementia were bedridden, making tasks such as repositioning and personal hygiene care particularly difficult. While some caregivers received occasional assistance from others when bathing or cleaning their care recipients, the majority had to manage these responsibilities alone: ‘*I’m tired, asking my children and grandchildren to help me with bathing him. But they don’t do it. Sometimes I feel hurt, I feel sad, and I even cry.’* (C1, age 70 yrs, wife).

### Theme 2: behavioral changes in PwD increase caregiver distress

#### The care recipient’s aggression placed significant strain on caregivers

Some caregivers reported experiencing physical or verbal violence from their care recipients, intensifying their stress. One caregiver noted that family members distanced themselves from the care recipient due to her aggressive behavior. Aggression also posed challenges in medical settings, as illustrated by one caregiver’s account: *‘When the nurse took her blood sample, she resisted forcefully. She shouted, bit the nurse and even hit the doctor’* (C5, age 52, daughter). One caregiver speculated that her mother’s aggression stemmed from an inability to express her needs. However, while some caregivers struggled with their recipients’ hallucinations, they did not find them particularly distressing.

#### Caregivers experienced exhaustion due to disruptions in the sleep patterns of PwD

Some caregivers struggled with their loved ones’ altered sleep cycles, particularly nighttime wakefulness. Prolonged sleep deprivation led to both physical and psychological strain. One caregiver described resorting to medication to manage her husband’s sleep disturbances: *‘I have to buy sleeping pills from the pharmacy store for him. If not, he will not sleep and will wander around and rummage through cabinets all night. No one can endure that’* (C1, age 70, wife).

### Theme 3: caregivers faced challenges in seeking help

#### Caregivers accepted their responsibilities but faced challenges in seeking support from family members

Most caregivers viewed caregiving as an obligation, often shaped by cultural or familial expectations. As one caregiver stated, *‘I am the youngest child and single, it is my duty to care for my mom*.’ Some caregivers assumed the role because they had more free time than other family members. Many caregivers endured the burdens of caregiving in silence, believing that asking for help would be an imposition. Even when caregivers sought assistance, family members were often unwilling to provide support.

#### Caregiving was perceived as a female responsibility

Some female caregivers expressed the belief that caregiving tasks were inherently suited to women, leading them to refrain from seeking help from male family members. Additionally, some caregivers assumed that male family members lacked the necessary skills or experience to provide adequate care, as noted by a female caregiver: *‘In general, three sons cannot care for my mom. They don’t know anything*.’ (C15, age 65 yrs, daughter).

#### Hiring a professional care worker was not considered a viable option by family caregivers

Many caregivers believed that a hired professional would not provide the same level of care and attention as a family member. Additionally, financial constraints made this option unattainable for some caregivers: ‘*I don’t have money to hire a person to care for my sister, so I have not thought about this*.’ (C19, age 60 yrs, younger sister).

### Theme 4: family caregivers sacrificed health, finance, and time for their caring roles

#### Family caregivers made significant personal sacrifices, often at the expense of their own health

Many caregivers reported a decline in their well-being over time, experiencing chronic pain, sleep deprivation, and weight loss. Some even neglected their own medical needs, including forgetting to take prescribed medications. Additionally, the physical demands of caregiving increased their risk of musculoskeletal problems: ‘*In recent years, I have more illnesses, more back pain, and frequent muscle strains*’ (C5, age 52 yrs, daughter).

#### Caregivers sacrificed their time and professional careers to fulfill their caregiving responsibilities

Some had to leave well-paying jobs to care for their loved ones, prioritizing their role as caregivers over financial stability. Many believed that their loved ones had limited time left, which motivated them to spend as much time as possible with them. As a result, caregivers willingly gave up leisure activities and often felt guilty when considering personal time. One caregiver expressed this sentiment, stating, ‘*If I go out and enjoy myself, I will leave my mom alone at home. She will feel pitiful, so I don’t go out’* (C13, age 53, daughter).

### Theme 5: family caregivers faced emotional challenges in caring

#### Caregivers experienced frustration and emotional distress

Their stress was exacerbated by the progressive decline of their loved ones, often conflicting with their expectations or hopes. As one caregiver expressed, ‘*Besides physical strain, I also have emotional strain*’ (C14, age 35, daughter).

#### Caregivers struggled with guilt and self-doubt

They blamed themselves for not providing adequate care for their loved ones. One caregiver believed that her mother’s injury was her fault: ‘*She fell because of me. Every time I remember that moment, I can’t hold back my tears*’ (C17, age 58, daughter).

#### Caregivers experienced fear and anxiety regarding the comorbidity of PwD

Some felt overwhelmed when managing pressure injuries, while others constantly worried about their loved ones falling. One caregiver expressed, ‘*If she falls, she will die. Now she is very old, so her bones are getting brittle. If her femur breaks, she will die*.’ (C15, age 65 yrs, daughter). Caregivers were also concerned about potential infections in their care recipients that could lead to hospitalization, particularly pneumonia and urinary tract infections: ‘*Normally, my mother often gets urinary tract infections. Her recent admissions were due to urinary tract infection and pneumonia*’ (C9, age 49 yrs, daughter).

### Theme 6: family caregivers had limited knowledge about dementia and lacked essential caring skills

#### Caregivers had limited knowledge about dementia

They viewed dementia as a normal part of aging rather than as a medical condition, believing that memory loss was simply a natural consequence of growing older: ‘*At the age of 75 or older, older people tend to lose their memories significantly*’ (C7, age 45, son). In contrast, some caregivers were confused about why their loved one’s cognitive and functional abilities continued to decline over time.

#### The information and skills related to caregiving were mainly derived from their personal experiences or online resource searches

Some caregivers reported that their caregiving practices relied on trial and error, while others turned to the internet, particularly through Google, for guidance. One caregiver expressed her uncertainty about administering medication: ‘*The biggest fear is when I give her medicine. Sometimes I do not know what they are, so I have to search for them online*’ (C16, age 50, daughter).

#### Essential caring skills for PwD

Caregivers were not equipped with adequate caring skills for an individual with dementia. ‘*I would like to know how to care for and feed a person with dementia. No one guides me on how to deal with a person with dementia*’ (C13, age 53 yrs, daughter).

## Discussions

Our study provides an in-depth exploration of the experiences and challenges faced by family caregivers of PwD. Through the interviews, we found that caregivers struggled with daily tasks, such as feeding, bathing, and maintaining hygiene for their loved ones. In particular, they encountered significant difficulties in caring for those who were incontinent or bedridden. These challenges are especially common among individuals with severe dementia, placing both physical and psychological strains on family caregivers [30]. Providing assistance with activities of daily living for PwD is a time-consuming and demanding task [31]. Therefore, training in everyday care skills is essential for family caregivers of PwD to enhance the quality of dementia care [32].

Behavioral and psychological symptoms in PwD contribute significantly to caregiver exhaustion. According to the caregivers, aggression and sleep disturbances were the most distressing symptoms. Care recipients exhibited verbal or physical aggression, such as swearing or hitting, while their disrupted sleep patterns led to severe sleep deprivation for caregivers. Some caregivers identified sleep disturbances as the most challenging aspect of providing care. These findings align with existing literature, which indicates that behavioral and psychological symptoms are among the most stressful aspects of dementia caregiving [33]. Such symptoms not only accelerate cognitive and physical decline in PwD but also impose significant physical and psychological burdens on caregivers [32]. While some caregivers managed their loved ones’ hallucinations and illusions, they viewed these symptoms as a normal part of aging.

Our study also highlights the profound sacrifices family caregivers made in terms of their health, time, and finances to fulfill their caring roles. We observed that self-care was often neglected by caregivers with many reporting experiencing weight loss, muscle strain, and joint pain and an increased risk of developing chronic diseases. Feelings of guilt prevented caregivers from engaging in leisure activities while some even left high-paying jobs to fulfill their caregiving roles. While some caregivers wished to spend every possible moment with their loved ones due to their limited time left, others held hope for improvement. These findings underscore the need for dementia education programs that enhance both caregiving and self-care skills, ultimately benefiting both caregivers and care recipients [34,35]. Furthermore, greater awareness of dementia progression and the caregiving journey is crucial for preparing families for the challenges ahead [36].

Despite these challenges, family caregivers often struggle to seek help. Many viewed caregiving as their sole responsibility, a belief that may be influenced by Taoist philosophy [37], which emphasizes filial duty. As a result, they endured significant physical and psychological strain without seeking assistance. Some feared inconveniencing other family members, while others, particularly female caregivers, refrained from seeking help from male relatives due to the perception that caregiving is a woman’s responsibility. Consequently, female caregivers often refrain from seeking help from male family members. This belief is rooted in traditional gender roles and male chauvinism in Vietnamese culture [38], which further increases the burden on female caregivers. Encouraging both men and women to participate in caregiving through targeted training and support programs could help alleviate this imbalance [39,40].

Hiring in-home caregivers was not a viable option for families due to financial constraints. Many caregivers had left paid employment, reducing household income and making professional care unaffordable. Others believed that hired caregivers would not provide the same level of care as family members. This perception may stem from the shortage of trained dementia caregivers in Vietnam’s healthcare system, highlighting gaps in available dementia care resources [13]. Addressing these gaps through trained and volunteer caregiver programs could strengthen support systems for dementia family caregivers.

The interviews revealed that family caregivers were particularly concerned about comorbidities in PwD, particularly falls, pressure injuries, and infections. Frequent falls were a major challenge, as dementia is a well-established risk factor for falls [41]. Cognitive and physical decline, including visual and auditory impairments, wandering, and behavioral and psychological symptoms, further increased fall risk [42]. In the later stages of dementia, when PwD become bedridden, the risk of pressure injuries and infections rises [43]. Many family caregivers felt unprepared to prevent or manage pressure injuries, emphasizing the need to integrate this topic into caregiver training programs [44]. Recurrent hospital readmissions due to infections, particularly pneumonia and urinary tract infections, were another major concern. Infections are a leading cause of mortality among PwD [45,46] with risks including dysphagia, poor oral hygiene, incontinence, prolonged immobility, and comorbidities such as diabetes mellitus [46,47]. These findings emphasize the need for targeted education and training programs that equip family caregivers with the knowledge and skills to effectively manage these common health complications.

### Study limitations

This study was conducted among family caregivers of PwD in Ho Chi Minh City, the largest city in Vietnam. Consequently, our findings may not fully capture the experiences of caregivers in rural areas, where access to healthcare services is more limited, and caregiving challenges may be even greater. Another limitation of our study is the absence of participant checking, which may have limited the opportunity for participants to verify our interpretations. However, we sought to enhance the credibility of our findings through real-time participant validation during the interviews.

Despite these limitations, our study highlights the urgent need to integrate dementia education to be integrated into dementia care policies in Vietnam and other Eastern LMICs. Educational programs should encompass dementia knowledge, practical caregiving skills, help-seeking strategies, and physical and emotional self-care for caregivers. Furthermore, developing support resources, such as respite care, accessible information platforms (e.g. online resources or support groups), and social workers specializing in dementia care will be essential to strengthening the support system for both caregivers and PwD. In clinical practice, greater emphasis should be placed on equipping caregivers of PwD with the skills to manage comorbidities, particularly in the later stages of dementia. Training should include fall prevention, pressure injury care, and infection prevention, ensuring caregivers are better prepared to meet the complex needs of PwD.

## Conclusions

Overall, the study highlights the challenges and needs of family caregivers of PwD. In addition to the daily difficulties of providing care, caregivers struggled to seek help and prioritize self-care, potentially influenced by cultural factors, such as Taoism or male chauvinism. They also expressed concerns about managing the comorbidities in PwD. These aspects should be incorporated into caregiver training programs and interventions aimed at reducing caregivers’ strain. Addressing these challenges will not only enhance care quality for PwD but also improve the well-being of both caregivers and care recipients, particularly in resource-limited settings like Vietnam.

## Appendix

**Table:**

Main points of discussion	
1	How is it that you came to be involved in caregiving? (i.e. was it simply your responsibility, or was there a family discussion about who would be the primary caregiver?)
2	Can you tell me the impact that caregiving has had on you? (i.e. physically, psychologically, socially, financially/employment, marital relationship, etc.)
3	What type of care do you provide to the person with dementia? (e.g. physical, food, bathing, toileting, financial, etc.) What challenges have you found when providing this specific type of care? (e.g. if you take care of the person with nutritional needs, what has been the most challenging part of this task?)
4	Can you tell me the greatest problems you’ve faced when finding out a loved one has dementia? (i.e. what are/were the main issues you face? What are the main issues you need help with to manage? e.g. knowledge/skills, etc.)
5	Where do you often search for information and support? (e.g. online, from peers/family, information provided by healthcare professionals/peers/family, etc.) How helpful have these resources been? What gaps of information and support have you recognized? (e.g. if you turn to your healthcare professional the most, how helpful has this been? Have there been times when your healthcare professional wasn’t able to give you the information or support you needed?)
6	What do you want or need to help in your caregiving role? (i.e. if you were to design a new information source for someone like yourself, what things would you focus on?) What information, skills, or support do you want to receive presently? (e.g. dementia disease, skills of caregiving, etc.)
7	What are your thoughts on developing an online resource for caregivers of individuals with dementia? (i.e. how useful is it?)
8	Do you have any advice that you would give us in developing an online resource for caregivers of people with dementia? (i.e. how would you like information presented? e.g. written, videos, storytelling, etc.)
